# Supplementary material for: Early-adult methionine restriction reduces methionine sulfoxide and extends lifespan in Drosophila
Source: Nat Commun. 2023 Dec 5;14:7832. doi: 10.1038/s41467-023-43550-2 (PMC10698029; doi:10.1038/s41467-023-43550-2)
Supplement: Supplementary file 1 — Supplementary Information [file 41467_2023_43550_MOESM1_ESM.pdf]

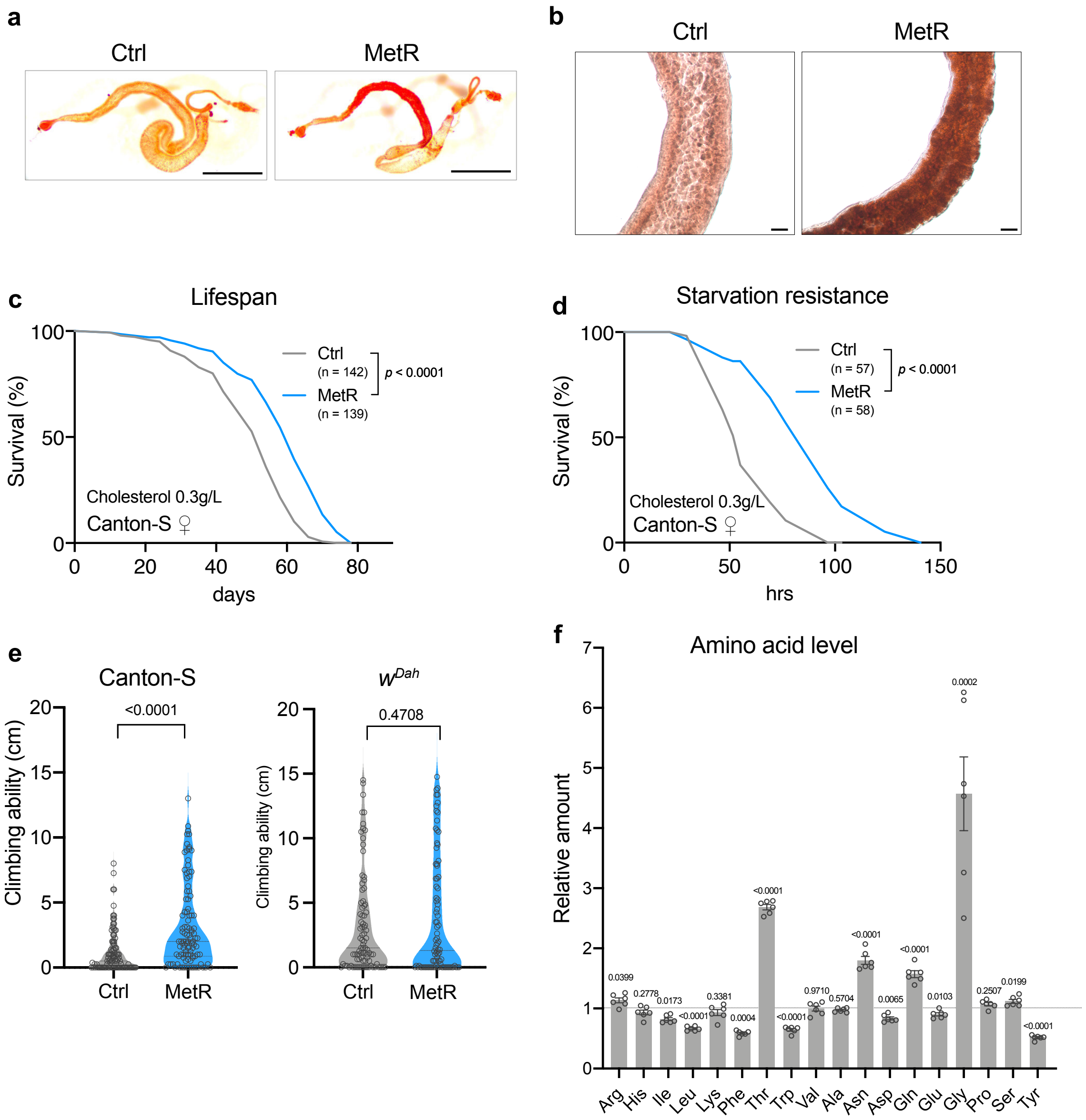

Supplementary Fig. 1 Phenotypic analysis of methionine-restricted flies.

a, b, Oil red O staining of female guts of Canton-S flies fed with or without a methionine-restricted diet for nine days. Whole gut (**a**) or magnified view of the anterior midgut (**b**). Scale bar: 1 mm (**a**), or 100 μm (**b**). **c**, Lifespans of female Canton-S flies fed with or without a methionine-restricted diet that contained three times as much cholesterol. Sample sizes (n) are shown in the figure. For the statistics, a log-rank test was used. **d,** Survivability of female Canton-S flies upon complete starvation after feeding with or without a methionine-restricted diet that contained three times as much cholesterol for a week. Sample sizes (n) are shown in the figure. For the statistics, a log-rank test was used.

e, Climbing abilities of female Canton-S or w^{Dah} flies fed with or without a methionine-restricted diet for four weeks. **f,** Quantification of amino acids other than methionine in female w^{Dah} flies upon methionine restriction. The relative amount of each amino acid upon methionine restriction compared to the control diet is shown. n = 6. For the statistics, a two-tailed Student's *t* test was used. For the graph, the mean and SEM are shown. Data points indicate biological replicates. Source data are provided as a Source Data file.

a

GO term "determination of adult lifespan"

Gene Name	baseMean	log2 fold change
dawdle(daw)	195	2.15
CG8846 gene product from transcript CG8846-RA(Thor/4E-BP)	12164	2.09
Methionine sulfoxide reductase A (MsrA)	1529	2.06
Peptidoglycan recognition protein LF(PGRP-LF)	42	1.90
period(per)	568	1.85
midkine and pleiotrophin 1(miple1)	21	1.48
CG10383 gene product from transcript CG10383-RB(CG10383)	1750	1.15
bunched(bun)	6576	0.99
puckered(puc)	1544	0.94
Death regulator Nedd2-like caspase(Dronc)	253	0.75
pointed(pnt)	667	0.71
CG42663 gene product from transcript CG42663-RB(CG42663)	639	0.64
Histone deacetylase 1(HDAC1)	546	-0.61
Coenzyme Q biosynthesis protein 2(Coq2)	141	-0.76
Phosphoribosylamidotransferase(Prat)	254	-0.77
Catalase(Cat)	13162	-0.80
CG31148 gene product from transcript CG31148-RA(CG31148)	3351	-0.93
Heat shock protein 68(Hsp68)	90	-1.89

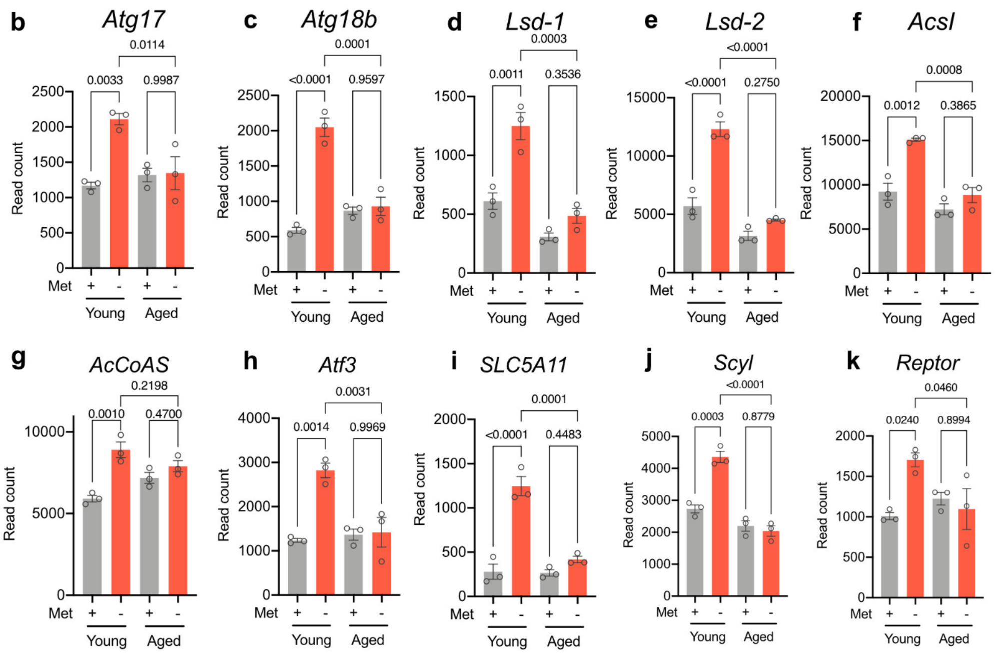

Supplementary Fig. 2 Transcriptomic responses to methionine restriction in young and aged flies.

a, List of genes termed “determination of adult lifespan” by Gene Ontology analysis. **B-k,** Read counts of genes induced by methionine restriction at a young age from the RNAseq analysis. N = 3. For the statistics, one-way ANOVA with Holm-Šídák’s multiple comparison test was used. For all graphs, the mean and SEM are shown. Data points indicate biological replicates. Source data are provided as a Source Data file.

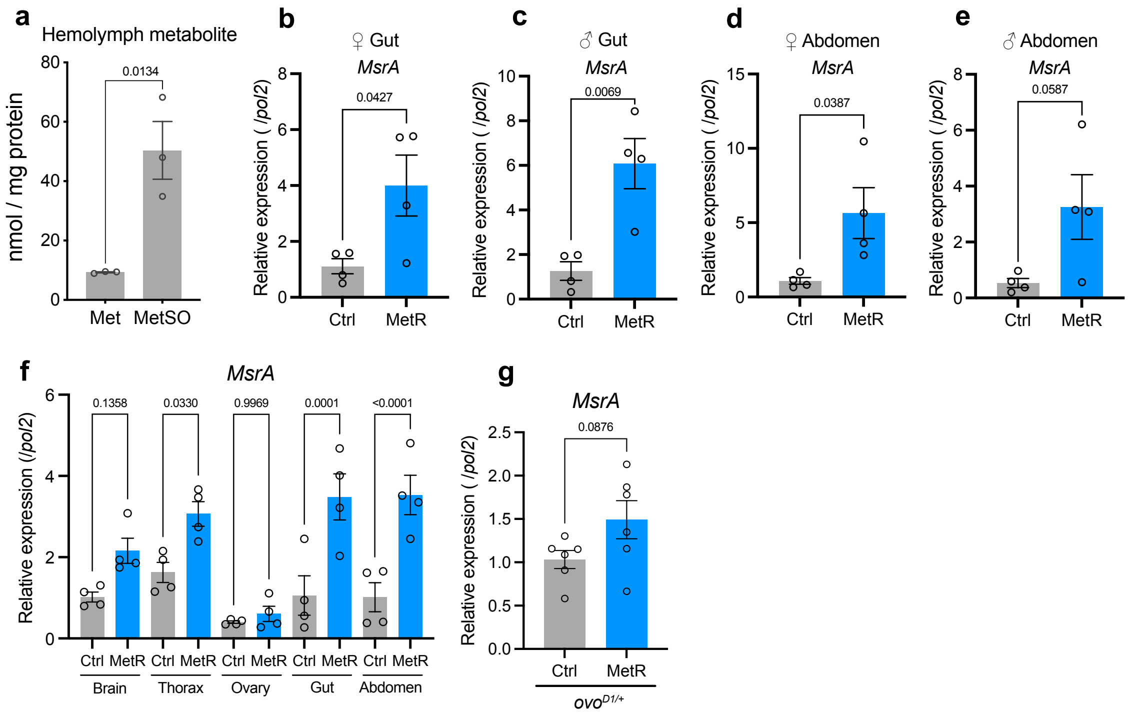

Supplementary Fig. 3 *MsrA* induction upon methionine restriction.

a, Quantification of Met and MetSO in the hemolymph of female Canton-S flies that were fed with a standard yeast-based diet for four days post-eclosion. n = 3. For the statistics, a two-tailed Student's *t* test was used. **b-e,** Quantitative RT–PCR analysis of *MsrA* expression levels in female guts (**b**), male guts (**c**), female abdomens (**d**) and male abdomens (**e**) of Canton-S flies fed with or without a methionine-restricted diet for three days. n = 4. For the statistics, a two-tailed Student's *t* test was used. **f**, Quantitative RT–PCR analysis of *MsrA* expression in various tissues of female Canton-S flies fed with or without a methionine-restricted diet for three days. n = 4. For the statistics, one-way ANOVA with Holm-Šídák's multiple comparison test was used. **g**, Quantitative RT–PCR analysis of *MsrA* expression in female guts of $ovo^{D1/+}$ fed with or without a methionine-restricted diet for three days. n = 6. For the statistics, a two-tailed Student's *t* test was used. For all graphs, the mean and SEM are shown. Data points indicate biological replicates. Source data are provided as a Source Data file.

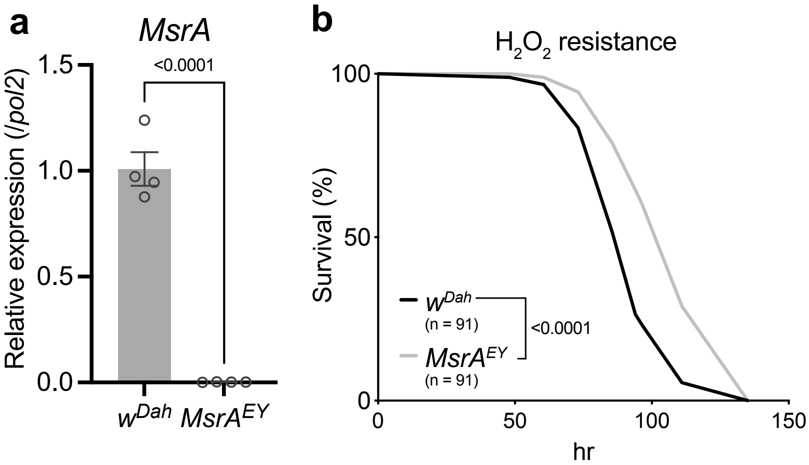

Supplementary Fig. 4 Characterisation of the *MsrA* mutant.

a, Quantitative RT–PCR analysis of *MsrA* in the whole bodies of w^{Dah} or $MsrA^{EY05753}$ flies. n = 4. For the statistics, a two-tailed Student's *t* test was used. **B**, Survivability of female flies of w^{Dah} or $MsrA^{EY05753}$ upon 3% H_2O_2 treatment. Sample sizes (n) are shown in the figure. For the statistics, a log-rank test was used. For the graph, the mean and SEM are shown. Data points indicate biological replicates. Source data are provided as a Source Data file.

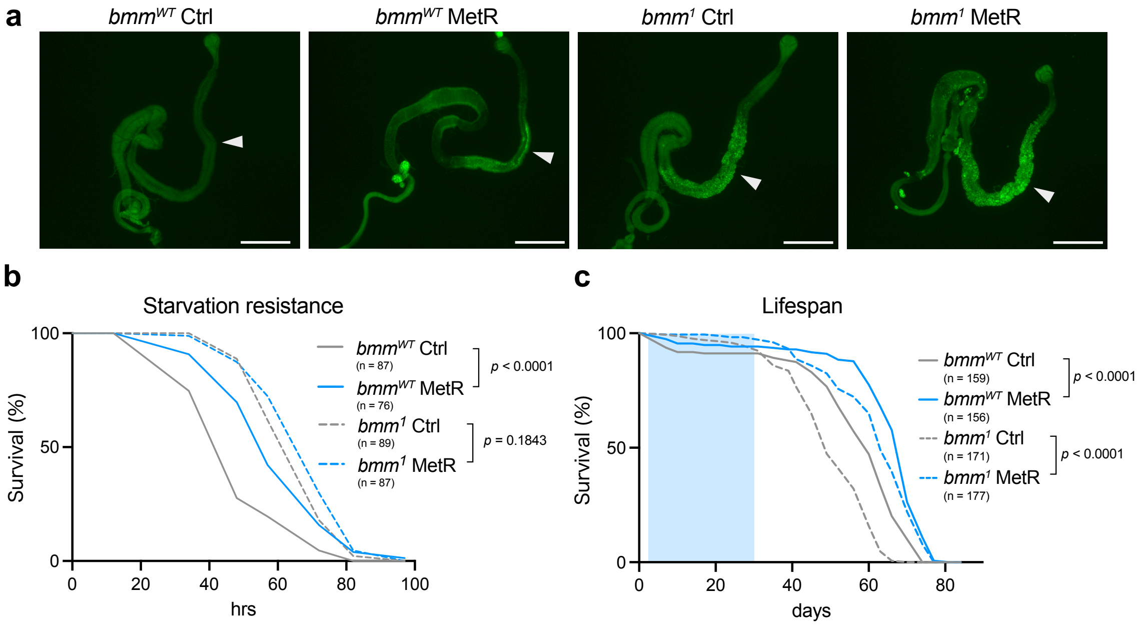

Supplementary Fig. 5 Contribution of lipid metabolism to lifespan extension upon methionine restriction.

a, Lipid staining of the female guts of *bmm*^{wt} and *bmm*^{1} flies fed with or without a methionine-restricted diet for one week using LipixTOX. Scale bar: 1 mm. Arrowheads indicate lipid accumulation. **b,** Survivability of female *bmm*^{wt} and *bmm*^{1} flies upon complete starvation after feeding with or without a methionine-restricted diet for one week. Sample sizes (n) are shown in the figure. For the statistics, a log-rank test was used. **c,** Lifespans of female flies of *bmm*^{WT} and *bmm*^{1} fed with or without a methionine-restricted diet in early life. Sample sizes (n) are shown in the figure. For the statistics, a log-rank test was used. Source data are provided as a Source Data file.

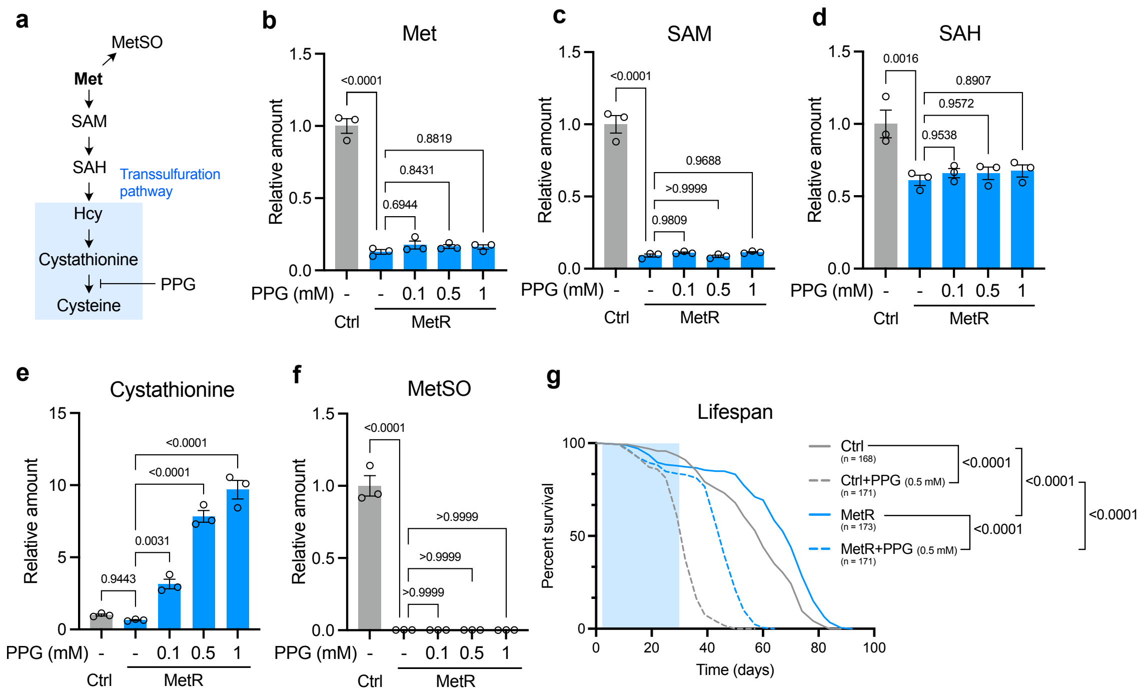

Supplementary Fig. 6 Inhibition of Transsulfuration pathway did not abrogate lifespan extension upon methionine restriction.

a, Methionine metabolic and transsulfuration pathways, which can be inhibited by propargylglycine (PPG). **b-f,** Quantification of methionine metabolites and their oxidative products upon methionine restriction. n = 3. For the statistics, one-way ANOVA with Holm-Šídák's multiple comparison test was used. **g,** Lifespans of female Canton-S flies fed with or without a methionine-restricted diet supplemented with 0.5 mM PPG. Sample sizes (n) are shown in the figure. For the statistics, a log-rank test was used. For all graphs, the mean and SEM are shown. Data points indicate biological replicates. Source data are provided as a Source Data file.

a

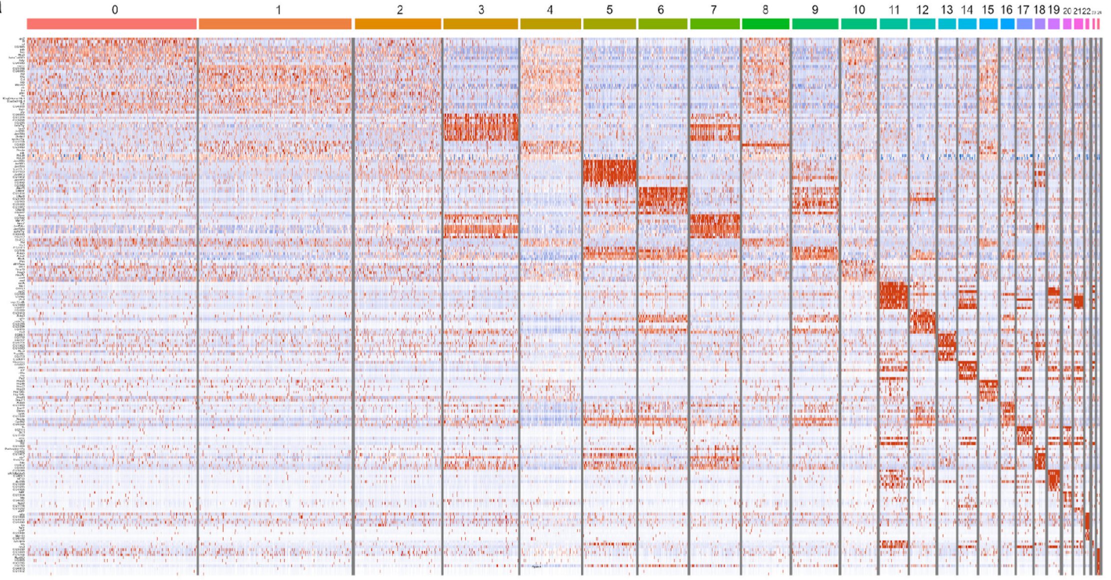

b

Cluster	Cell type	Key genes
0	EB/ISC	Dl, Tet, esg
1	EB1	klu, esg, Tet
2	EB2	klu, esg, Tet
3	aEC1	alphaTry, betaTry
4	EB3	klu, esg, Tet
5	LFC	mag, CG10472, Jon99Fii
6	pEC1	LManV, LManVI
7	aEC2	alphaTry, betaTry, Bace
8	EB4	klu, esg, Tet
9	pEC2	LManVI
10	ISC	Dl, HmgD, hoip, Awd
11	AstA-EE	AstA, AstC, CCHa1
12	pEC3	nub, Gs2, zetaTry
13	mEC	Vha16-1, Vha13, CG5767
14	Orcokinin-EE1	Orcokinin, AstC, IA-2
15	EB5	klu, esg, Tet
16	pEC4	nub, Npc2e
17	IA-2/Tk-EE	IA-2, Tk, DH31
18	copper/iron cell	MtnD, CG5399
19	AstC-EE	AstC, CCHa2
20	NPF-EE	NPF, IA-2
21	CCHa2-EE	CCHa2, IA-2
22	unknown	whe, CG34212
23	Orcokinin-EE2	Orcokinin, AstC, IA-2
24	cardia	Pgant4, Muc68D

c

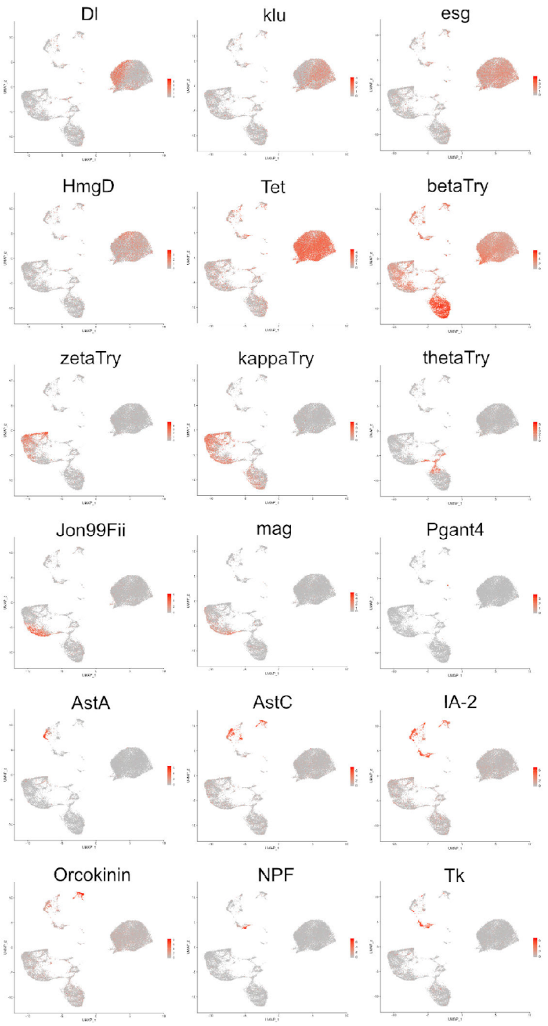

Supplementary Fig. 7 Cell type clustering analysis and marker gene expression in each cluster.

a, Heatmap of clustered marker genes from the results of single-cell RNAseq of the *Drosophila* female midgut. **b,** Cell type names in clusters. **c,** UMAP plot of characteristic marker genes.

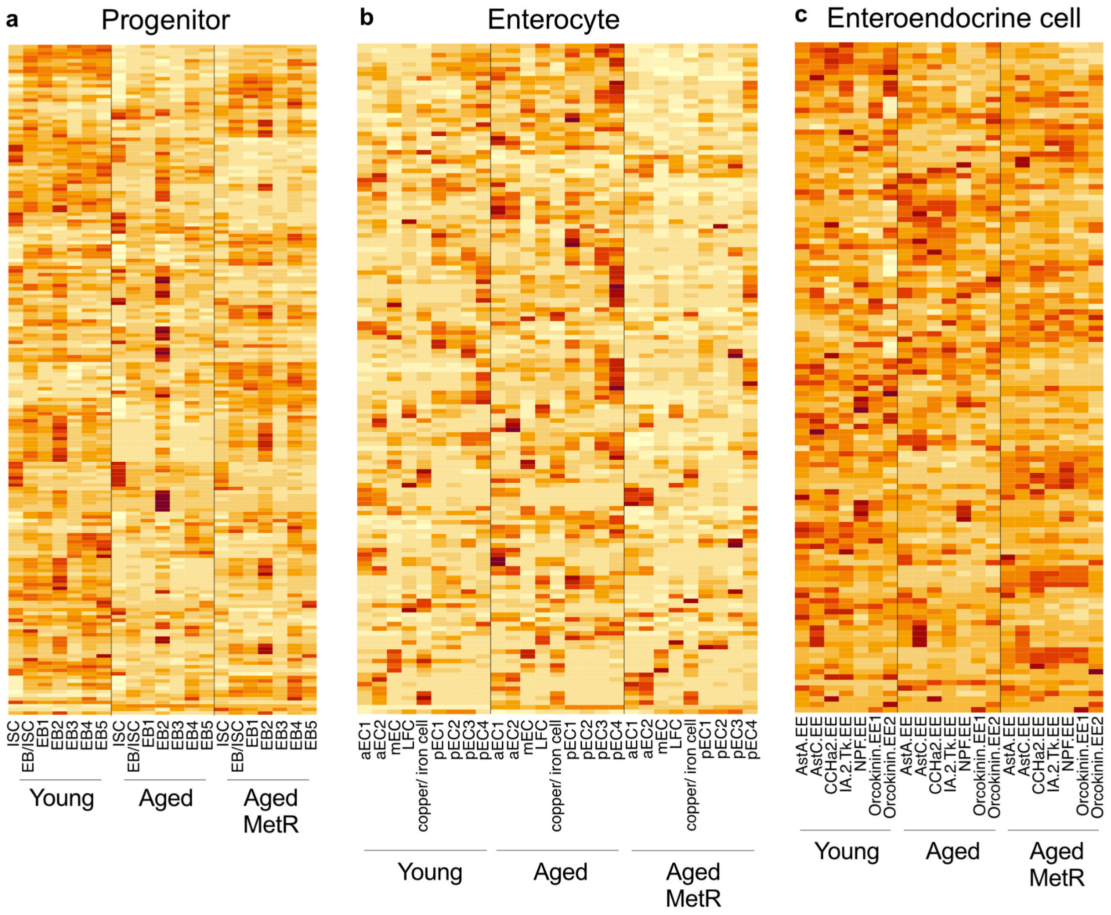

Supplementary Fig. 8 Heatmap analysis of DEGs from single-cell RNAseq.

a-c, Heatmap of DEGs in each cell type classified as progenitor cells (**a**), enterocytes (**b**), and enteroendocrine cells (**c**) upon methionine restriction with ageing. Source data are provided as a Source Data file.

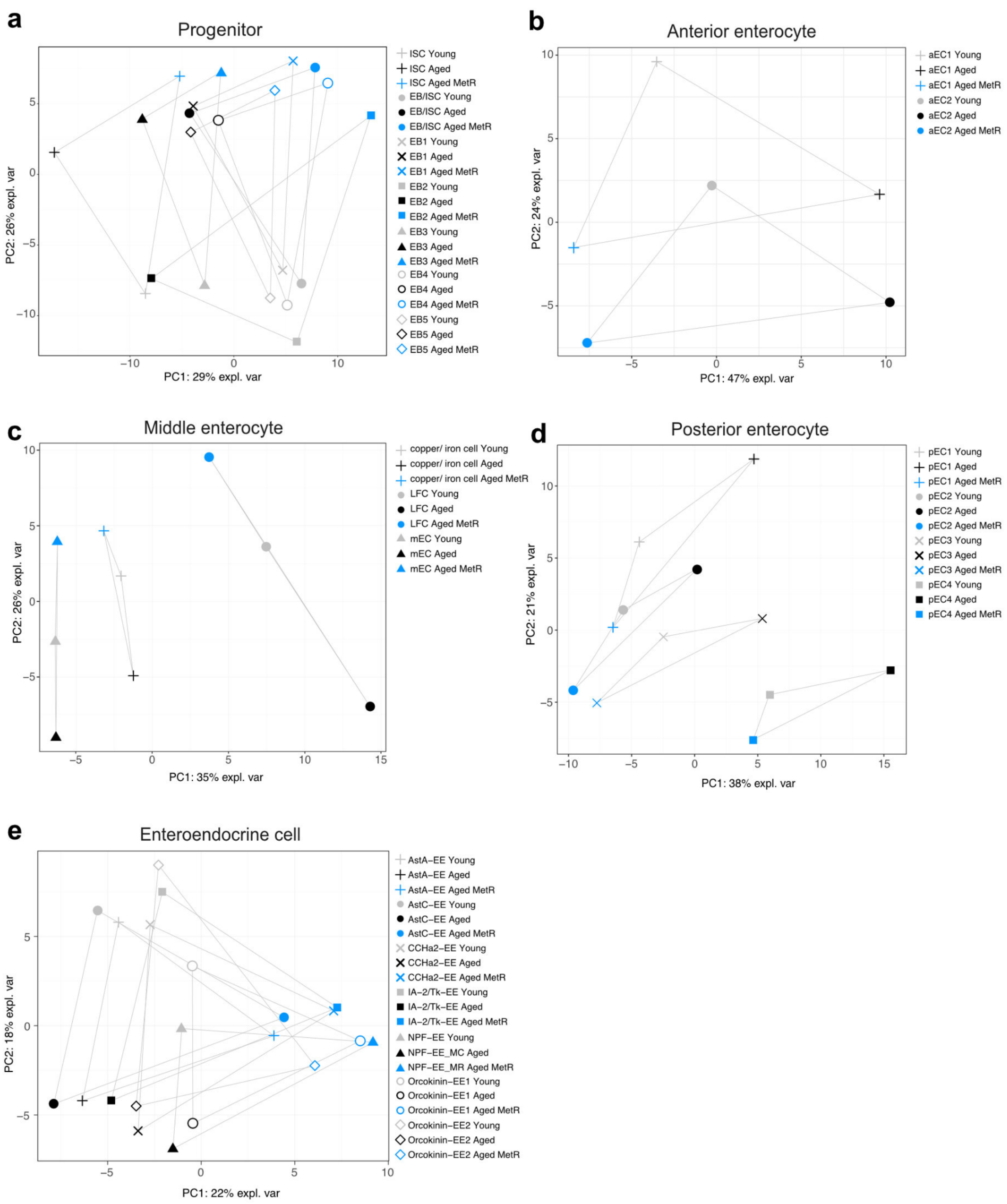

Supplementary Fig. 9 PCA of single-cell RNAseq results upon methionine restriction.

a-e, PCA of DEGs in each cell type classified as progenitor cells (**a**), anterior enterocytes (**b**), middle enterocytes (**c**), posterior enterocytes (**d**), and enteroendocrine cells (**c**) upon methionine restriction with ageing.

Supplementary Table 1. Cox PH analysis for w^{iso31} and w^{Dah} female flies.

	Dependent variable:	
	Age (days)	
	w^{iso31}	w^{Dah}
	coefficient (SE)	coefficient (SE)
Timing of diet treatment	-0.156 (0.125)	-0.048 (0.119)
MetR diet	-0.753^{***} (0.130)	-0.524^{***} (0.120)
Timing of diet treatment: MetR diet	0.589^{***} (0.178)	0.191 (0.167)
Observations	528	576
Score (Logrank) Test (df = 3)	38.854^{***}	27.841^{***}

$^{***}p$ <0.001

Supplementary Table 2. Cox PH analysis for *MsrA*^{EY05753} flies compared to w^{Dah} flies.

	Dependent variable:
	Age (days)
	coefficient (SE)
Genotype	-0.367^{***} (0.109)
MetR diet	-1.240^{***} (0.110)
Genotype: MetR diet	1.264^{***} (0.153)
Observations	703
Score (Logrank) Test (df = 3)	143.7^{***}

*** p <0.001

Supplementary Table 3. Cox PH analysis for *bmm*^{1} flies compared to *bmm*^{WT} flies.

	Dependent variable:
	Age (days) coefficient (SE)
Genotype	0.946*** (0.117)
MetR diet	-0.790*** (0.116)
Genotype: MetR diet	-0.695*** (0.160)
Observations	663
Score (Logrank) Test (df = 3)	248***

*** p <0.001
