# Supplementary material for: Early-adult methionine restriction reduces methionine sulfoxide and extends lifespan in Drosophila
Source: Nat Commun. 2023 Dec 5;14:7832. doi: 10.1038/s41467-023-43550-2 (PMC10698029; doi:10.1038/s41467-023-43550-2)
Supplement: Supplementary file 3 — Description of Additional Supplementary Files Document [file 41467_2023_43550_MOESM3_ESM.pdf]

Description of Additional Supplementary Files

Supplementary Data 1: DEG list obtained by RNAseq analysis of young or aged gut after complete Met depletion

Supplementary Data 2: DEG list obtained by time-series 3’RNAseq analysis of gut after MetR

Supplementary Data 3: DEG list of each cell cluster obtained by single cell RNAseq analysis

Supplementary Data 4: Ingredients and procedures to make holidic medium

Supplementary Data 5: All mass spectrometry data

Supplementary Data 6: R code for Cox PH analysis
