# Supplementary material for: Early-adult methionine restriction reduces methionine sulfoxide and extends lifespan in Drosophila
Source: Nat Commun. 2023 Dec 5;14:7832. doi: 10.1038/s41467-023-43550-2 (PMC10698029; doi:10.1038/s41467-023-43550-2)
Supplement: Supplementary file 10 — Reporting Summary [file 41467_2023_43550_MOESM10_ESM.pdf]

nature portfolio

Corresponding author(s): Fumiaki Obata
Last updated by author(s): Sep 25, 2023

Reporting Summary

Nature Portfolio wishes to improve the reproducibility of the work that we publish. This form provides structure for consistency and transparency in reporting. For further information on Nature Portfolio policies, see our Editorial Policies and the Editorial Policy Checklist.

Statistics

For all statistical analyses, confirm that the following items are present in the figure legend, table legend, main text, or Methods section.

n/a	Confirmed	
☐	☒	The exact sample size (*n*) for each experimental group/condition, given as a discrete number and unit of measurement
☐	☒	A statement on whether measurements were taken from distinct samples or whether the same sample was measured repeatedly
☐	☒	The statistical test(s) used AND whether they are one- or two-sided<br>*Only common tests should be described solely by name; describe more complex techniques in the Methods section.*
☒	☐	A description of all covariates tested
☐	☒	A description of any assumptions or corrections, such as tests of normality and adjustment for multiple comparisons
☐	☒	A full description of the statistical parameters including central tendency (e.g. means) or other basic estimates (e.g. regression coefficient) AND variation (e.g. standard deviation) or associated estimates of uncertainty (e.g. confidence intervals)
☐	☒	For null hypothesis testing, the test statistic (e.g. *F*, *t*, *r*) with confidence intervals, effect sizes, degrees of freedom and *P* value noted<br>*Give P values as exact values whenever suitable.*
☒	☐	For Bayesian analysis, information on the choice of priors and Markov chain Monte Carlo settings
☒	☐	For hierarchical and complex designs, identification of the appropriate level for tests and full reporting of outcomes
☒	☐	Estimates of effect sizes (e.g. Cohen's *d*, Pearson's *r*), indicating how they were calculated

Our web collection on statistics for biologists contains articles on many of the points above.

Software and code

Policy information about availability of computer code

Data collection	No unpublished code to collect the data. Detailed data collection methods were described in Methods.
Data analysis	No unpublished code to analyse the data. Detailed data analysis methods were described in Methods. BlueBee Platform (LEXOGEN), FastQC (v0.11.9), Trimmomatic (v0.39), Hisat2 (v2.1.0), subread (v1.6.5), DESeq2 (v1.26.0) were used for RNAseq analysis. Cell Ranger (v.5.0.1), Seurat (v3.1.0), mixOmics package (3.14), ggplot2 package (3.3.5) were used for single cell RNAseq analysis. OASIS 2 was used to perform log-rank test for lifespan analysis. Survival package (3.4-0) and car package (3.1-1) were used for Cox PH analysis. GraphPad Prism 8 and 9 was used for data presentation and statistical analysis.

For manuscripts utilizing custom algorithms or software that are central to the research but not yet described in published literature, software must be made available to editors and reviewers. We strongly encourage code deposition in a community repository (e.g. GitHub). See the Nature Portfolio guidelines for submitting code & software for further information.

Data

Policy information about availability of data

All manuscripts must include a data availability statement. This statement should provide the following information, where applicable:

- Accession codes, unique identifiers, or web links for publicly available datasets
- A description of any restrictions on data availability
- For clinical datasets or third party data, please ensure that the statement adheres to our policy

All relevant data are available from the authors upon request. The NGS data generated in this study have been deposited in the DDBJ and GEO under accession numbers DRA013585, DRA013644, and GSE198149. The genome datasets used in this study was BDGP6.22 for RNAseq analysis the guts of young or old flies upon 24 hr Met restriction, BDGP6 for 3' RNAseq analysis of guts after 6 hours, 2 weeks, or 4 weeks of Met restriction, BDGP6.28 for single-cell transcriptome analysis. Source data are provided with this paper.

Research involving human participants, their data, or biological material

Policy information about studies with human participants or human data. See also policy information about sex, gender (identity/presentation), and sexual orientation and race, ethnicity and racism.

Reporting on sex and gender	N/A
Reporting on race, ethnicity, or other socially relevant groupings	N/A
Population characteristics	N/A
Recruitment	N/A
Ethics oversight	N/A

Note that full information on the approval of the study protocol must also be provided in the manuscript.

Field-specific reporting

Please select the one below that is the best fit for your research. If you are not sure, read the appropriate sections before making your selection.

☒ Life sciences ☐ Behavioural & social sciences ☐ Ecological, evolutionary & environmental sciences

For a reference copy of the document with all sections, see nature.com/documents/nr-reporting-summary-flat.pdf

Life sciences study design

All studies must disclose on these points even when the disclosure is negative.

Sample size	Sample size was determined empirically. A similar sample size was used in previous papers (DOI: 10.1038/ncomms9332, DOI: 10.7554/eLife.62335).
Data exclusions	No data were excluded.
Replication	NGS analyses were not repeated due to limitation of budget, but the leading conclusion was confirmed by qPCR.<br>All other data were confirmed, at least twice, by the same or closely-related experiments.
Randomization	Animals were allocated ramdomly to different dietary conditions.
Blinding	No blind tests were implemented because the same investigator set up the experiments, collected samples, and analysed the data. However, the samples and data were collected under identical conditions.

Reporting for specific materials, systems and methods

We require information from authors about some types of materials, experimental systems and methods used in many studies. Here, indicate whether each material, system or method listed is relevant to your study. If you are not sure if a list item applies to your research, read the appropriate section before selecting a response.

Materials & experimental systems

n/a	Involved in the study
☒	☐ Antibodies
☒	☐ Eukaryotic cell lines
☒	☐ Palaeontology and archaeology
☐	☒ Animals and other organisms
☒	☐ Clinical data
☒	☐ Dual use research of concern
☒	☐ Plants

Methods

n/a	Involved in the study
☒	☐ ChIP-seq
☒	☐ Flow cytometry
☒	☐ MRI-based neuroimaging

Animals and other research organisms

Policy information about studies involving animals; ARRIVE guidelines recommended for reporting animal research, and Sex and Gender in Research

Laboratory animals	The following fly stocks were used in this study. All adult flies that eclosed within one day were collected and maintained for an additional two days for maturation and mating on the standard diet. Flies were then sorted by sex and maintained for indicated period.<br><br>Canton-S<br>wiso3l(from Dr.Alex. Gould)<br>wDah (doi: 10.1371/journal.pone.0004067)<br>da-GeneSwitch (from Dr. Monnier Veronique)<br>NP1-Gal4, tub-Gal8Ots (Jiang et al., 2009)<br>UAS-foxo-RNAi (BDSC, 27656)<br>bmm[WT] (doi: 10.1016/j.cmet.2005.04.003., 10.1016/j.ibmb.2020.103498)<br>bmm[1] (doi: 10.1016/j.cmet.2005.04.003., 10.1016/j.ibmb.2020.103498)<br>ovo[D1] (BDSC, 1309)<br>UAS-lacZ (Kyoto, 106500)<br>UAS-foxo (BDSC, 9575)<br>MsrA[EY05753](BDSC, 16671)
Wild animals	The study did not use wild animals.
Reporting on sex	We used mated female flies unless otherwise stated.
Field-collected samples	The study did not include samples from field-collected samples.
Ethics oversight	No ethical approval was required.

Note that full information on the approval of the study protocol must also be provided in the manuscript.
